# Corrosion on Copper Induced by Biodiesel Surrogates in the Gas Phase: The Effect of the C=C Double Bond

**DOI:** 10.3390/ma18184395

**Published:** 2025-09-20

**Authors:** Fabiola Vergara-Juarez, Emilio Hernandez-Medina, Jesus Porcayo-Calderon, Macdiel Emilio Acevedo-Quiroz, Jose Trinidad Perez-Quiroz, Alfredo Quinto-Hernandez

**Affiliations:** 1 Departamento de Ingeniería Química y Bioquímica, Tecnológico Nacional de México/Instituto Tecnológico de Zacatepec, Calzada Tecnológico 27, Zacatepec 62780, Morelos, Mexico; fabizvj@gmail.com (F.V.-J.); l19091208@zacatepec.tecnm.mx (E.H.-M.); macdiel.aq@zacatepec.tecnm.mx (M.E.A.-Q.); 2 Departamento de Ingeniería Química y Metalurgia, Universidad de Sonora, Hermosillo 83000, Sonora, Mexico; jesus.porcayo@unison.mx; 3 Instituto Mexicano del Transporte, Km. 12 Carretera Estatal No. 431, “El Colorado-Galindo”, San Fandilla, Pedro Escobedo 76703, Queretaro, Mexico; jtperez@imt.mx

**Keywords:** biodiesel, surrogate, corrosion, copper, unsaturation, thermogravimetric loss, electrochemical impedance spectroscopy, gas-phase

## Abstract

The C=C double bond is a typical feature in biodiesel components associated with their physicochemical behaviors, including metal degradation. In this work, copper was exposed to the gas-phase atmospheres of Methyl Hexanoate (MH) at 145 °C and 25% Methyl Trans-3-Hexenoate in Methyl Hexanoate (MT3H in MH) at 158 °C during 1000 h, representing saturated and unsaturated thermal degradation environments of biodiesel surrogates. FTIR, ^1^H NMR, and GC-MS were used to characterize the chemical changes in the gas-phase atmospheres, whereas SEM allowed us to inspect the copper surfaces. Weight loss assays enabled the estimation of corrosion rates for copper exposed to HM and MT3H in MH atmospheres of 3.81 ± 1.27 and 5.08 ± 1.27 μm/year, respectively. Electrochemical measurements (linear polarization resistance (LPR) and electrochemical impedance spectroscopy (EIS)) were used to evaluate the corrosion behavior of copper using aqueous extracts of condensed compounds from gas-phase degraded environments. Our electrochemical results indicate that similar corrosion processes occur in both extracts, remaining nearly unchanged with increasing exposure time. A porous layer of corrosion products on copper revealed that it is more active in the products generated with the MT3H in MH extract, suggesting the significant impact of the C=C bond on copper deterioration.

## 1. Introduction

Over the last few decades, replacing fossil fuels with biofuels has been of great interest, as it can reduce some undesirable environmental impacts, such as greenhouse gas emissions [1]. Biodiesel is a biofuel that wholly or partially replaces conventional diesel, with a zero-carbon footprint [2,3]. Typically, biodiesel is obtained from vegetable oils and animal fats [4], as well as from other sources, such as microalgae lipids [5] and sewage sludge [6], through the process of transesterification [7]. The result of this reaction is a mixture of saturated and unsaturated methyl esters with long alkyl chains, with between 15 and 20 or more carbon atoms [8]. Motivations for employing biodiesel as a transportation fuel include the minimal amount of sulfur compounds released into the atmosphere [9], the limited need for modifications to current compression-ignition engines [10], its biodegradability, and its environmentally less-hazardous properties [11]. However, economic and technical drawbacks have a negative impact when using biodiesel, as it is more costly and exhibits poorer oxidative stability, cold flow properties, and higher corrosion tendency to metals than diesel [12]. Innovations in biodiesel require overcoming such drawbacks via experimental or theoretical work. However, as the chemical constituents of biodiesel are large molecules, their study becomes challenging, using either of these approaches. Investigations into biodiesel components can be simplified using functionally similar molecules with lower molecular weights that serve as surrogates [13,14]. Surrogate esters provide insight into the impact of individual biodiesel components, as they can reproduce the physical and chemical properties of the real alkyl esters present in this biofuel [15].

For example, several studies have been conducted using surrogates to analyze the effects of the degree of unsaturation (C=C bonds) and the alkyl chain length of biodiesel on engine performance, including combustion and soot production. Unsaturated surrogate esters have facilitated the identification of essential intermediate species (hydroperoxy alkyl-ester radicals, alkyl and alkyl-ester radicals) in biodiesel combustion [16], unveiling the influence of the C=C double bond on the formation of various emissions [17]. Regarding soot formation, studies focused on unsaturation [18] and alkyl chain length [19] of biodiesel surrogates have enabled the identification of soot precursors in combustion experiments, including benzene and acetylene [20]. Acetylene has a role in controlling nitrogen oxide (NO_x_) emissions [18], a major contributor to atmospheric pollution. Additionally, biodiesel surrogates have been used in the development of reliable kinetic models that incorporate related reactions to biodiesel, such as oxidation, unimolecular dissociation, atomic hydrogen, and radical abstractions [21]. Methyl formate [22], methyl acetate [23], methyl butanoate [24], ethyl formate [25], and ethyl propionate [26] are some of the most commonly used esters to understand the reactions listed above.

To the best of our knowledge, reports on the corrosive properties of biodiesel surrogates on metals, such as those used in engine manufacture, remain unavailable. Furthermore, a review reveals that investigations of corrosion caused by biodiesel in the gas phase, or its surrogates, on metal surfaces are limited. Gas-phase corrosion is relevant, since biodiesel components are transferred as vapors when a spray is produced during engine operation. One should expect that using biodiesel surrogates enables the assessment of corrosion, given the molecular characteristics of biodiesel components or the products formed during engine operation. Finally, given their simplified molecular geometry, biodiesel surrogates may provide insight into reaction mechanisms (such as oxidation and auto-oxidation) induced by biodiesel on metal surfaces [27,28] at a fundamental level.

Methyl Hexanoate (CH_3_(CH_2_)_4_COOCH_3_, or MH) and Methyl Trans-3-Hexenoate (CH_3_ CH_2_CH=CHCH_2_COOCH_3_, or MT3H) are two suitable candidates for identifying corrosive characteristics within the biodiesel surrogate group, due to their molecular size [29]. MH is an intermediate in the combustion of long-chain methyl esters [30], and it has been utilized in kinetic modeling [31,32], identification of soot precursors [32], and determination of autoignition of biodiesel [16]. The impact of the C=C double bond near this methyl ester, specifically the Methyl Trans-3-Hexenoate (MT3H), has also been assessed in kinetic modeling [33]. Thus, the present study focuses on the corrosion phenomenon of copper using MH in the gas phase, and MT3H as its unsaturated surrogate. The deterioration of copper exposed to these surrogate esters is reported using mass loss and electrochemical measurements, including linear polarization resistance and electrochemical impedance spectroscopy. We utilized gas-phase environments to gain insight into the effect of the C=C bond in biodiesel components on the corrosion behavior of vaporized biodiesel on copper.

## 2. Materials and Methods

### 2.1. Materials

Methyl Hexanoate (CAS 106-70-7) and Methyl Trans-3-Hexenoate (CAS 2396-78-3) were of analytical grade and purchased from Sigma-Aldrich (St. Louis, MO, USA). These reagents were used without further purification. The copper coupons used in this work had a purity of 99.99%.

### 2.2. Weight Loss

Copper coupons (17.5 mm × 17.5 mm × 3 mm) were gradually ground using silicon carbide abrasive paper with grit sizes P100, P240, and P600. They were then washed with distilled water and subsequently cleaned ultrasonically for 15 min in an acetone bath. The copper exposure area for each coupon was estimated considering a 2 mm diameter hole drilled near the edge, where each coupon was suspended for immersion. In each test, three coupons were exposed independently to gas phase atmospheres of Methyl Hexanoate (MH) and a mixture of 25% Methyl Trans-3-Hexenoate in Methyl Hexanoate (MT3H in MH). The tests were conducted using a hydrothermal reactor with a 50 mL volume of liquid HM or MT3H in MH, which had been previously degassed. The metallic samples were suspended inside the reactor, avoiding contact with any biodiesel surrogate. Once the air above the liquid was displaced, the reactor was hermetically closed and heated under atmospheric pressure at 145 °C for HM (boiling point HM, 149 °C) [34] and 158 °C for MT3H in HM (boiling point MT3H, 168 °C) [34] for 1000 h. After the test time, the reactors were cooled down to room temperature, and the metal samples were then removed and cleaned, according to ASTM G1-03, to measure weight loss [35]. The measurement accuracy of the weight loss was 0.001 g, and the instrument used was a Shimadzu Balance Model TX223L (Shimadzu Corporation, Kyoto, Japan). The corrosion rate was determined in accordance with ASTM G31-21 [36]. The average and standard deviation values of the corrosion rate were calculated from the weight loss of the three copper coupons used in each assay.

### 2.3. Analysis of Chemical Modifications

The biodiesel surrogate samples recovered both before and after the tests were characterized using Fourier-transform infrared (FTIR) analysis, ^1^H nuclear magnetic resonance (^1^H NMR), and gas chromatography coupled with mass spectrometry (GC-MS). FTIR spectra were recorded in the range 4000−800 cm^−1^ using an FTIR-ATR Perkin Elmer Spectrum One spectrometer (PerkinElmer, Shelton, CT, USA), during 16 scans at a resolution of 4 cm^−1^. We used a Nuclear Magnetic Resonance (NMR) instrument, Bruker Avance™ III HD 400 MHz (Bruker Corporation, Billerica, MA, USA), whose spectra were obtained at 400 MHz with CDCl_3_ as the solvent. For GC-MS analysis, measurements were obtained with an Agilent Technology 6890 gas chromatograph (Agilent Technologies, Santa Clara, CA, USA) and a 5973 N mass spectrometer. During its operation, helium was dosed at a flow rate of 1 mL min^−1^, and the injection volume was 20 µL (CHCl_3_, HPLC grade). The apparatus employed an HP-5MS capillary nonpolar column connected to an ion trap detector operating at 70 eV in electron impact mode. We compare our GC-MS results with the NIST/EPA/NIH Mass Spectral Library (version 1.7a/ChemStation).

### 2.4. Surface Analysis

The surface morphology of the copper samples was examined after exposure to the gaseous environment (HM or MT3H in HM) with scanning electron microscopy (SEM). Here, we used a Hitachi S5500 electron microscope equipped with a Duo-STEM detector (Hitachi, Tokyo, Japan) at 10 kV (ASTM E986) [37]. All SEM microphotographs are shown with a magnification of 500×.

### 2.5. Electrochemical Measurements

Electrochemical measurements consisted of linear polarization resistance (LPR) (ASTM G59-97) [38] and electrochemical impedance spectroscopy (EIS) (ASTM G106-89) [39]. The tests were performed in a three-electrode cell using a Gamry (Interface 1010E) potentiostat/galvanostat/ZRA (Gamry Instruments, Warminster, PA, USA). The reaction area of the working electrode was 1 cm^2^, and it was polished with abrasive paper prior to each assay. The aqueous fraction of the thermally degraded biodiesel surrogates was used as the corrosive medium for the electrochemical measurements. Such an aqueous fraction, or aqueous extract, was obtained as follows. As described in Section 2.2. Weight Loss, 50 mL of degassed HM was thermally degraded in a hydrothermal reactor for 500 h at 145 °C. In a separate assay, 50 mL of degassed MT3H in HM was also subjected to the same conditions, but at 158 °C. No copper was present in the degradation of corrosive media during both of these assays. The resulting samples were cooled down to room temperature and then vigorously mixed for 24 h with an equal volume of distilled water to obtain the soluble fraction that causes the corrosion process. As a comparison, distilled water was also used as a corrosive medium.

Electrochemical impedance spectroscopy (EIS) testing for copper immersed in biodiesel surrogate products was performed for 24 h by applying a 20 mV alternating current perturbation in the frequency range of 100 kHz to 0.01 Hz. Linear polarization measurements were obtained at one-hour intervals for 25 h by polarizing the electrode ±15 mV with respect to its potential, at a sweep rate of 10 mV/min.

Due to the high resistivity of the solution and to mitigate the problems associated with data acquisition, it was necessary to decrease the resistance between the electrodes of the electrochemical cell, as previously suggested [40,41]. Therefore, platinum wires were used as the reference and counter electrodes were placed over the working electrode (a copper-made plate) to cover the sample surface, while maintaining a 2 mm separation between them. Unlike conventional reference electrodes, the platinum reference electrode is well-suited for complex measurements [42,43]. Using platinum as a reference electrode for impedance measurements has been reported elsewhere [44,45]. We considered it suitable in this study as a reference electrode, due to the medium’s high resistivity.

## 3. Results

### 3.1. Chemical Analysis of Degraded Biodiesel Surrogates

**Figure 1:**
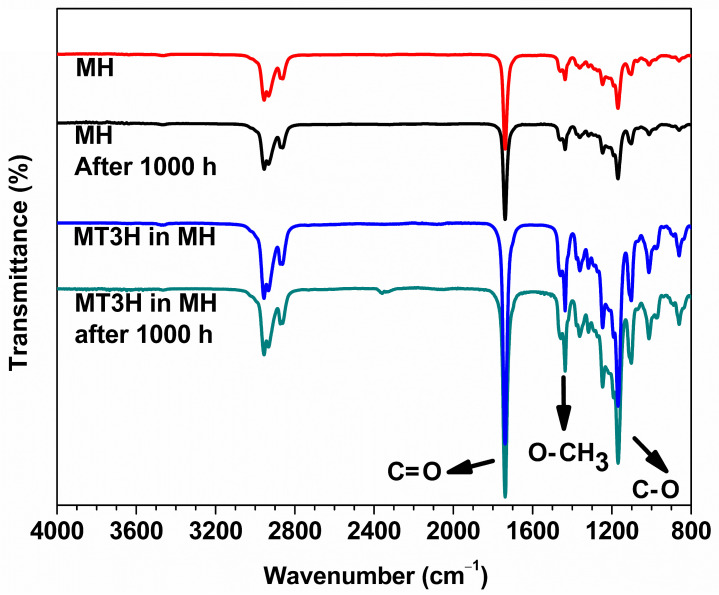
FTIR spectroscopy of biodiesel surrogates after being exposed to copper samples at the corresponding degradation temperatures. FTIR spectra of corrosive media included: from top to bottom, Methyl Hexanoate (MH) at 0 h (red line) and after exposition of 1000 h (black line), and 25% Methyl Trans-3-Hexenoate in Methyl Hexanoate (MT3H in HM) at 0 h (blue line) and after exposition of 1000 h.

FTIR, ^1^H NMR, and GC-MS analysis of the biodiesel surrogate samples provide evidence of the chemical changes that occurred during our assays. Figure 1 presents the FTIR spectra of MH (red continuous line) and MT3H in HM (blue continuous line), which were recorded before the weight loss tests. We also include the FTIR spectra of the condensed samples of MH (black continuous line) and MT3H in HM after 1000 h of exposure at the corresponding volatilization temperatures. All the spectra show absorption signals characteristic of methyl esters. At ~1740 cm^−1^, the C=O (carbonyl) stretch signal is present, while the O-CH_3_ (methoxy) and C-O (ester) stretch signals are found at ~1435 and ~1168 cm^−1^, respectively [46]. As can be observed, all the spectra are similar. In comparison to the FTIR spectra recorded previously, the characteristic IR bands remain unchanged or unshifted after the weight loss tests, complicating the identification of chemical changes.

**Figure 2:**
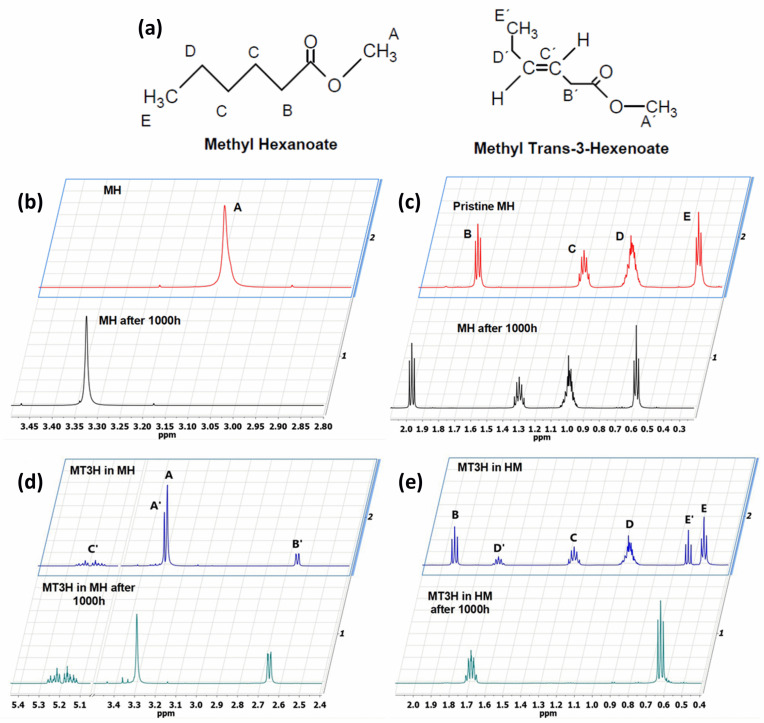
^1^H NMR Spectroscopy of biodiesel surrogates after being exposed to copper samples at the corresponding degradation temperatures. (**a**) Generic chemical structures and proton designation of Methyl Hexanoate and Methyl Trans-3-Hexenoate, NMR ranges included. (**b**) 3.45–2.80 ppm, and (**c**) 2.0−0.3 ppm for ^1^H NMR Spectra of Methyl Hexanoate (MH) at 0 h (upper panels, red line) and after exposition of 1000 h (lower panels, black line), and ^1^H NMR Spectra of 25% Methyl Trans-3-Hexenoate in Methyl Hexanoate (MT3H in HM) at 0 h (upper panels, blue line) and after exposition of 1000 h in the ranges of (**d**) 5.40−2.40 ppm, and (**e**) 2.0−0.4 ppm. Letters A–E indicate ^1^H NMR signals of Methyl Hexanoate, and A’–E’ indicate characteristic ^1^H NMR signals associated with Methyl Trans-3-Hexenoate.

The methyl esters collected in this work were also characterized by ^1^H NMR spectroscopy (Figure 2). To guide the interpretation of our ^1^H NMR spectra, generic structures of Methyl Hexanoate and Methyl Trans-3-Hexenoate are introduced in Figure 2a. Pristine MH exhibits a characteristic signal of the methoxy (CH_3_O) protons (Figure 2b, upper panels) observed as a singlet at 3.08 ppm (signal A), while the methylene (CH_2_) protons of carbon B are present at 1.76 ppm as a triplet (Figure 2c, upper panel, signal B). Other signals related to the aliphatic chain correspond to the methylene hydrogens of carbon C, which are found in the region 1.04–1.10 ppm as a quintuplet (signal C), and the hydrogens of carbon D present as a multiplet between 0.80 and 0.75 ppm (signal D). Finally, a triplet is centered at 0.36 ppm, attributed to the protons of the terminal methyl group (CH_3_).

The ^1^H NMR spectrum of the condensed MH after degradation (Figure 2b,c, lower panels) exhibited some changes compared to the pristine MH. The characteristic ^1^H NMR signals of the degraded MH undergo a chemical shift to a lower field. This result indicates that the protons in the degraded and condensed MH are less shielded than those in the pristine MH, due to the temperature’s effect on the degradation of the biodiesel surrogate. The NMR spectrum of degraded HM also suggests that the aliphatic chain remains undeprotonated, as the olefin proton signals are absent. However, this evidence implies a modification in the aliphatic chain, given the shift to a lower field. Figure 2d,e (upper panels) show the ^1^H-NMR spectrum of the pristine mixture of MT3H in HM. Here, characteristic signals of the two methyl esters, HM and MT3H, are observed.

The signals corresponding to the unsaturated ester undergo a high-field shift, while the ^1^H-NMR signals of the saturated ester remain similar to the description provided above. The chemical shift observed in the signals of MT3H is consistent with the protons present in the carbonate chain. The protons of the methoxy group characteristic of MT3H are found at 3.29 ppm (signal A’), whereas the allylic protons of carbons B’ and D’ are observed as a doublet of doublets at about 2.65 ppm and a multiplet at 1.64–1.70 ppm, respectively. The olefinic protons C’ (C=C double bond) are visible as a multiplet in the region of 5.11–5.26 ppm. Finally, CH_3_ protons (E’) are present in the high field region with a chemical shift of 0.60–0.63. No new signals were generated in this spectrum, and we concluded that the interaction between HM and MT3H in the mixture was of a physical nature.

Next, the mixture of MT3H in HM was degraded at 158 °C after 1000 h, and the spectrum obtained is shown in Figure 2d,e, once the mixture was condensed. The ^1^H NMR spectrum is placed in the lower panels. The ^1^H NMR signals of HM remain unobserved in this spectrum, suggesting that the ester has been completely converted. In contrast, the multiplet in the range of 5.11–5.26 ppm indicates that the C=C bond in MT3H, or an unsaturated product, might be preserved after thermal degradation. In fact, other ^1^H NMR signals of MT3H are also present [47]. As discussed later, this implies that a portion of the unsaturated ester remained unreacted or transformed into products producing the signal already described. Unsaturated fatty acids can auto-oxidize, producing hydroperoxides and/or aldehydes, which exhibit signals in the region of 6–10 ppm [48]. Due to the absence of these signals, it can be deduced that the mixture of substitutes was restricted from undergoing auto-oxidation.

GC-MS was used to investigate the formed and condensed species in the hydrothermal reactor, after the thermal degradation of the corrosive media MH and MT3H in MH. (See Table 1.) Before the weight loss tests, pristine HM and the mixture MT3H in HM were also identified via CG-MS, thus confirming the results obtained using FTIR and ^1^H NMR. Product 1 (Propanedioic acid, butyl-dimethyl ester), formed from HM at 145 °C, corroborates the interpretation of ^1^H NMR shown in the lower panel of Figure 2b,c.

Product 1 and MH must have similar ^1^H NMR spectra, due to symmetry arguments in the former compound. However, the tertiary carbon in Propanedioic acid, butyl-dimethyl ester, whose protons are denoted as a (Table 1), must also be identified with a triplet signal at a lower field. This feature is the primary difference between the two spectra, and is found at 1.97 ppm.

The resulting condensed products from the thermal degradation at 158 °C of the mixture MT3H in MH are 2-Pentenoic acid, 4-Methyl, Methyl ester (Product 2), Butanoic acid, 3-Methyl-2-Methylene, Methyl ester (Product 3), and 4-Pentenoic acid, 2-Acetyl-4-Methyl, Methyl ester (Product 4). Here, we also noted that unreacted MH remained unidentified via GC-MS after 1000 h of thermal degradation, a finding that was also observed in the ^1^H NMR study. Branched Products 2 to 4, identified by GC-MS, are also comparable with the ^1^H NMR spectrum of Figure 2d,e, lower panels. The compatibility of our ^1^H NMR and GC-MS results becomes apparent when considering that all NMR signals must be observed, unnecessarily, when analyzing a mixture of products. The larger the quantity of individual components to identify, the more complex the ^1^H NMR spectrum can be. In a mixture, we must expect that some signals might overlap or be obscured. To interpret the ^1^H NMR of the degraded MT3H in MH, we consider that all products are characterized by the group CH_3_OCO (methoxycarbonyl), whose protons a correspond to singlets in the range of 3.40 to 3.25 ppm. Also, protons b in the C=C double bond are detected in Figure 2d,e, lower panels, by the multiplet in the 5.1–5.3 ppm region, a typical range associated with unsaturated chains. The C=C double bond is evident in all condensed products in Table 1. Suppose the protons **c** in the tertiary carbon of the structure of Products 2 and 3 should be observed as multiplets; in that case, we noted a multiplet centered at 1.68 ppm. These same products are characterized by protons d, and neighboring carbons c, which we assigned to the doublet at about 0.90 ppm. Unfortunately, protons e, f, and g for Product 4 are obscured and unobserved in the corresponding ^1^H NMR spectrum (Figure 2d,e). Finally, signals at 0.65 and 2.65 ppm are associated with unreacted MT3H. The analysis of our ^1^H NMR spectra was confirmed using shift simulations in ChemDraw Professional 15.0.

### 3.2. Weight Loss

The average weight loss of the copper samples was calculated using the sample weights measured before and after exposure to the gas-phase biodiesel surrogates. The weight loss values were used to calculate the corrosion rate, using Equation (1) [36].

(1) $$\mathrm{Corrosion}\mathrm{rate}\;(\mu m/\mathrm{year})=\frac{K\times W}{A\times T\times D}$$

where the corrosion rate is estimated in μm/year, K is the corrosion constant (8.76 × 10^7^), W is the weight loss (g), A is the exposure area (cm^2^), T is the exposure time (hours), and D is the density of the metal, in this case (8.94 g cm^−3^).

The corrosion rate of copper exposed to HM vapors was 3.81 ± 1.27 μm/year, and for the MT3H in MH mixture, it was 5.08 ± 1.27 μm/year. The highest corrosion rate was obtained with MT3H in MH. To our awareness, gas-phase corrosion rates for HM, MT3H, or any other biodiesel surrogate remain unavailable, both for copper and for ASTM metals. Corrosion rates for biodiesel surrogates when the corrosive medium is liquid also remain unreported. Given the boiling point (b. p.) for Propanedioic acid, butyl-dimethyl ester is 219.6 °C [49]; the corrosion rate is associated exclusively with HM in our first assay in the gas phase, since Product 1 should have converted in the liquid phase. Regarding the MT3H in MH mixture, the products 2-Pentenoic acid, 4-Methyl, Methyl ester (b. p. = 84–86 °C) and Butanoic acid, 3-Methyl-2-Methylene, Methyl ester (b. p. = 139.0 °C) [49] have boiling points lower than that of the temperature for its thermal degradation. Consequently, these products might also be associated with the corrosion rate of the gaseous mixture. The product 4-Pentenoic acid, 2-acetyl-4-Methyl, Methyl ester (b. p. = 210 °C) must have been limitedly exposed in the gas atmosphere, and should have been produced in the liquid phase, too. For both corrosive media, the thermal conditions must have prevented the biodiesel surrogates from fragmenting, as pyrolytic conditions typically occur at higher temperatures and pressures. The result is consistent with previous research [50,51], which reports that C=C bonds lead to increased metallic corrosion. The gaseous compounds produced by MT3H in the MH mixture may be more reactive with the metal surface than those generated in MH, resulting in greater deterioration and an increased corrosion rate. Copper might have produced a CuO/CuCO_3_ film, followed by a Cu_2_O layer, which is produced after a prolonged exposure time [52]. In the case of the oxidation process, MH is primarily controlled by the C-H bonds of carbon α, regardless of temperature and pressure [29], resulting in the formation of hydroperoxides [31,53]. Other works [54,55,56] have demonstrated that copper can act as a catalyst for degrading methyl esters. Therefore, the generation of hydroperoxides produced in the MH oxidation process can be accelerated when they come in contact with copper oxides; the number of -COOH groups increases, which causes an increase in corrosion on the metal surface.

### 3.3. Morphological Analysis

**Figure 3:**
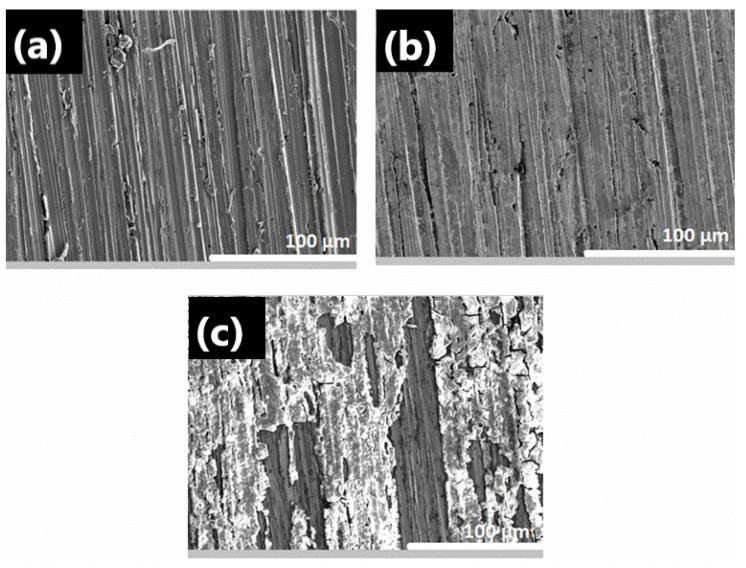
SEM microphotographs of copper specimens, (**a**) before thermal degradation assays, and after 1000 h of immersion in gas-phase corrosive media of (**b**) Methyl Hexanoate, and (**c**) 25% Methyl Trans-3-Hexenoate in Methyl Hexanoate. The magnification of all SEM images is 500×.

Figure 3 presents the surface appearance of copper samples, (a) before thermal degradation assays, and after exposure to the gas atmosphere of (b) Methyl Hexanoate and (c) 25% Methyl Trans-3-Hexenoate in Methyl Hexanoate during 1000 h. Figure 3a shows the surface appearance of copper, characterized by a clean surface and lines caused by smoothing with silicon carbide abrasive paper grade P600.

In Figure 3b, the surface appearance of copper exposed to gaseous MH shows a few corrosion products adhering to it. Here, the damage is evident, as the marks on the surface finish appear smoother, compared to those observed in Figure 3a. For the copper sample exposed to the MT3H in MH mixture (Figure 3c), corrosion products partially adhered to the surface are easily observed. The evidence suggests that the higher corrosivity of biodiesel surrogates is enhanced in the presence of unsaturation.

### 3.4. Electrochemical Measurements

**Figure 4:**
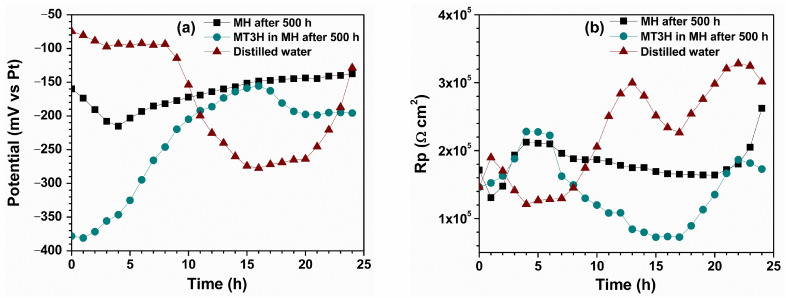
Variation of the (**a**) open circuit potential (OCP) and (**b**) polarization resistance (Rp) with respect to time of aqueous extracts obtained from Methyl Hexanoate (MH, solid black squares), and 25% Methyl Trans-3-Hexenoate in Methyl Hexanoate (MT3H in MH, solid green circles), after 500 h of thermal degradation, as well as distilled water, as a reference.

The variation in the open-circuit potential (OCP) value and polarization resistance (Rp) for copper in the aqueous extracts from the condensed samples of MH and the MT3H in MH blend is shown in Figure 4. In distilled water, copper exhibited the most noble potential OCP values during the first 10 h of gas immersion, and subsequently transitioned to more active values, until 20 h of immersion. See Figure 4a.

At the end of the test, the OCP values tended to increase, which may be attributed to the formation and growth of a protective oxide layer on the copper surface. The behavior of copper immersed in the aqueous extract of MH shows more active values than those observed in distilled water. In the first 4 h of immersion, the potential values shifted to more active values, and subsequently showed an increasing trend until the end of the test. This behavior may be associated with an initial stage of metallic dissolution and the subsequent formation of a protective layer on the copper surface. For the copper immersed in the aqueous fraction of MT3H in MH, the most active OCP values were obtained at the beginning of the test, with a constant tendency to increase until 17 h of immersion, followed by a decrease and then a steady state until the end of the test. The OCP values from the aqueous fraction of MT3H in MH demonstrate the greater aggressiveness of the aqueous extract and the copper’s capacity to protect itself.

Regarding the polarization resistance values (Figure 4b), copper immersed in distilled water exhibited low Rp values during the first 10 h of immersion. Still, these values tended to increase until the end of the test. The Rp values obtained are the highest of the three media evaluated. Such evidence suggests the formation of a stable protective layer. The Rp values obtained in the aqueous extract of MH showed the most stable behavior, exhibiting an ascending trend in the first 4 h of the immersion test and subsequently decreasing slightly, only to increase again when the test ended. For the aqueous extract of the MT3H in the MH mixture, the Rp values were less stable. When the test began, the Rp values tended to increase over the first 4 h of immersion, then decrease until 17 h, and increase again thereafter. In general, the Rp values in this medium were the lowest, suggesting a more aggressive nature of the electrolyte and that the surface layers developed were less protective than those formed in the presence of distilled water.

**Figure 5:**
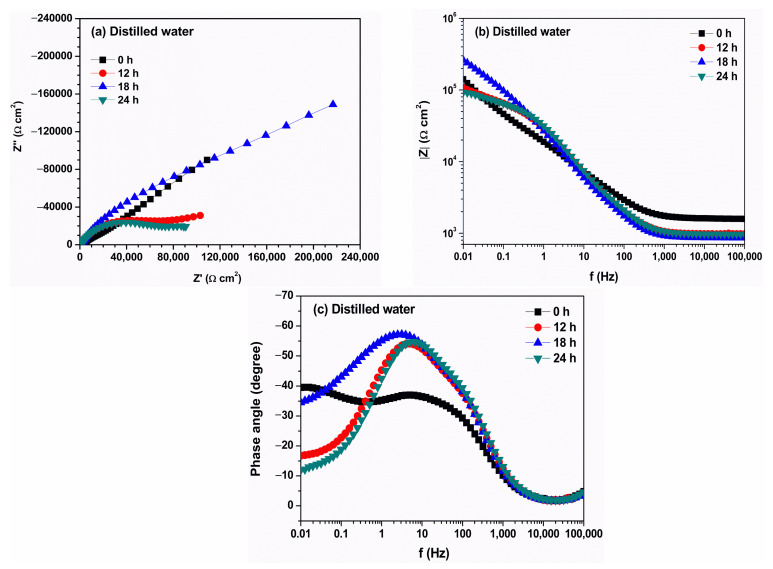
Evolution of electrochemical impedance spectra of copper immersed in distilled water according to (**a**) Nyquist, (**b**) Bode (Z module), and (**c**) Bode (phase angle). Diagrams were recorded for a duration of the test of 0 (black square), 12 (red circle), 18 (blue triangle), and 24 h.

**Figure 6:**
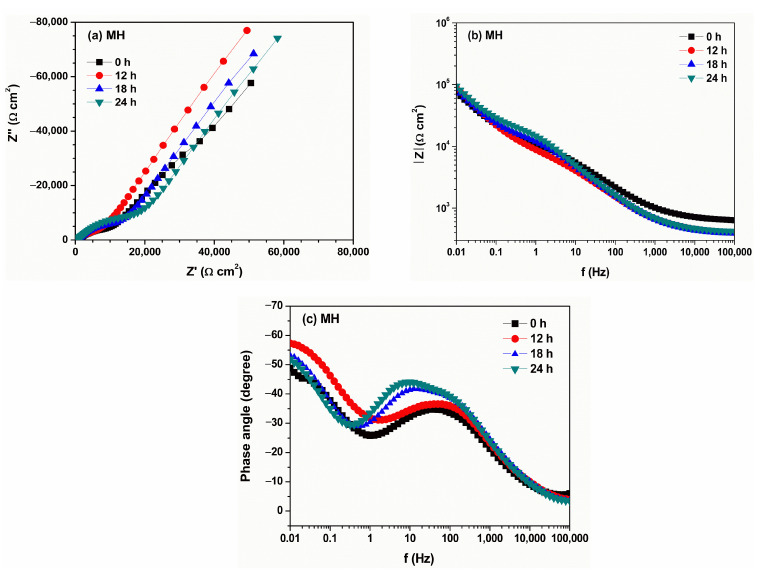
Evolution of electrochemical impedance spectra of copper immersed in aqueous extract of volatilized MH according to (**a**) Nyquist, (**b**) Bode (Z module), and (**c**) Bode (phase angle). Diagrams were recorded for a duration of the test of 0 (black square), 12 (red circle), 18 (blue triangle), and 24 h.

**Figure 7:**
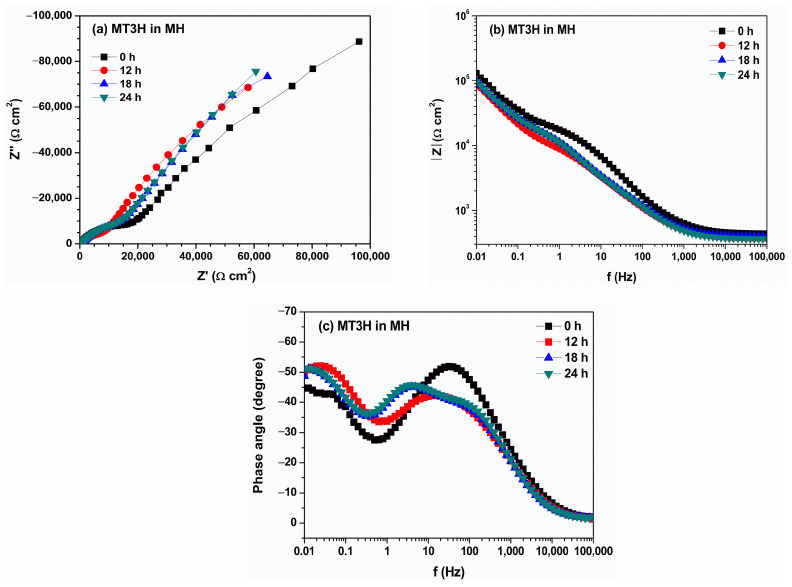
Evolution of electrochemical impedance spectra of copper immersed in aqueous extract of degraded MT3H in HM mixture according to (**a**) Nyquist, (**b**) Bode (Z module), and (**c**) Bode (phase angle). Diagrams were recorded for a duration of the test of 0 (black square), 12 (red circle), 18 (blue triangle), and 24 h.

EIS data for Nyquist and Bode analysis of copper in aqueous extracts of degraded biodiesel surrogates (HM and MT3H in HM), and distilled water as a reference, at different immersion times (0 to 24 h), are presented in Figure 5, Figure 6 and Figure 7. The Nyquist plot of copper in distilled water (Figure 5a) shows a single semicircle, which decreases at 12 h, and then increases at 18 h. At 24 h, the arc of the semicircle decreases, indicating greater activity on the surface. This behavior may be due to the rupture of the oxide layer formed. The Bode (Z module) plot (Figure 5b) shows a change in slope of the mean frequency, with the value of the impedance modulus decreasing at 12 h and then increasing at 18 h. In contrast, the characteristic phase-angle bell (Figure 5c) is more defined, and the amplitude increases with exposure time, reaching a maximum angle value of −57° at 18 h.

Figure 6 and Figure 7 show the EIS (Nyquist and Bode) plots obtained from measurements of the aqueous extracts of degraded MH and MT3H in MH, respectively. In both extracts, the shape of the spectra is similar, indicating that the corrosion process is identical in both samples. From the Nyquist diagram for the MH extract (Figure 6a), a capacitive arc is observed in the high- and intermediate-frequency regions, and the beginning of another one is evident in the low-frequency region. Features described in Figure 6a are corroborated by observing the Bode diagram in its impedance modulus format (Figure 6b), where a high-frequency well-defined plateau is evident above 10 kHz.

In the low-frequency region, a new slope is formed. The formation of the low-frequency plateau is limited, indicating that the impedance modulus is higher than the last recorded value. From the Bode diagram in its phase angle format (Figure 6c), a time constant is present in the high-frequency and intermediate-frequency regions, with the maximum phase angle increasing slightly over time. The presence of a second time constant is also observed, but in the low-frequency range, where its maximum value also increases slightly, over time. The first time constant corresponds to the capacitive arc observed in the Nyquist diagram, and this may be associated with the resistive–capacitive response of the metal surface. The second time constant may be related to adsorption–diffusion processes occurring on the metal surface.

The evolution of electrochemical impedance spectra obtained from the aqueous extract of degraded MT3H in MH (Figure 7) presents a behavior similar to that observed with the MH extract. The Nyquist diagram (Figure 7a) indicates the presence of a capacitive arc in the high- and intermediate-frequency regions, and the onset of another capacitive arc in the low-frequency region. The longer the exposure time, the larger the radius of the semicircle arc at medium and low frequencies. Therefore, the resistance to charge transfer is higher. Likewise, in the Bode (Z module) diagram (Figure 7b), the high-frequency plateau is defined above 10 kHz. In the low-frequency region, a new slope is observed without defining the formation of the low-frequency plateau. The Bode (phase angle) diagram indicates the presence of a time constant in the high-frequency and intermediate-frequency regions (Figure 7c), whose maximum phase angle decreases over time. In the low-frequency region, a second time constant is also observed, with its maximum value increasing slightly over time. The meaning of the constants is the same as previously discussed.

**Figure 8:**
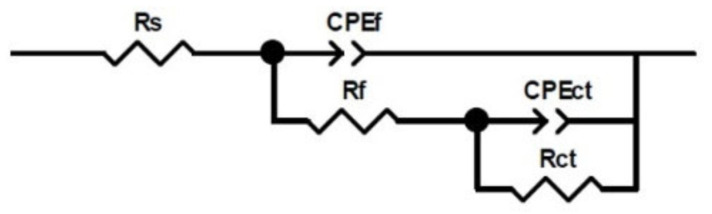
Equivalent electrical circuit for the impedance response of the copper–aqueous extract interface at different immersion times.

The impedance response of the electrochemical system can be similar to the impedance obtained from the electrical circuit scheme in Figure 8. The proposed circuit was used to obtain the main EIS parameters. Here, Rs is the solution resistance, Rct is the charge transfer resistance, and Rf is the resistance of the corrosion product film. CPEf and CPEct are constant phase elements used to indicate surface heterogeneities and account for frequency dispersion at the working electrode [57,58].

The impedance of a CPE is frequency-dependent and is defined by Equation (2).

(2) $$Z_{CPE}=\frac{1}{Y_{0}({\;jw)}^{n}}$$

where $Y_{O}$ is a proportionality factor; j = $\sqrt{-1}$, w is the angular frequency given by w=2πf; and n is a dimensionless parameter, ranging from 0 to 1. According to previous reports [59,60], the capacitance of a CPE can be obtained using the parameters $Y_{0\;}\;$ and α from Equation (3):

(3) Ci=( Y0iRi1−αi )1αi

Table 2 shows the evolution of the parameter values according to the proposed equivalent circuit (Figure 8). The goodness-of-fit for these values was found to be in excellent agreement with the obtained experimental data, with χ^2^ < 0.001. In addition, the results gathered were validated using Kramers–Kronig analysis [61] to demonstrate that the system fulfilled the required conditions (linearity, stability, and causality) in the impedance measurements. In all tests, the Rs values decrease with increasing exposure time, with the lowest values observed for the aqueous extract of MT3H in MH. Also, Rct values of the aqueous extract MT3H in MH are the lowest among the corrosive media, followed by the MH aqueous extract and distilled water, thus associating a tendency for Rct values with the presence of the C=C double bond. Such a tendency can be justified considering the second capacitive arc that appears in the low-frequency range, which is more evident in the extract MT3H in MH. The arc suggests a considerable interaction between the metal surface and the corrosive medium, where species adsorbed on the metal surface can form a layer of oxides that both protects and deteriorates the metal. Next, the corrosion product film strength, Rf, is significantly lower than the charge transfer resistance, Rct, in both aqueous extracts of MH and MT3H in MH, indicating that the corrosion products generated during exposure on the copper surface are insufficient to form a compact layer. This result is comparable with the polarization resistance, estimated as Rp = Rf + Rct, also for both aqueous extracts of MH and MT3H in MH. Here, Rp values for such extracts increase with increasing exposure time, while in distilled water they decrease. (See Figure 4b.) The tendency of the Rp values of those from the aqueous extracts and distilled water indicates the presence of soluble compounds in the extracts.

In both corrosive media, the capacitance value of the double layer (Cdl) decreases over 24 h with respect to its initial value, due to a decrease in the local dielectric constant and an increase in the thickness of the electrical double layer [62]. However, an increase in the Cdl value is observed (18 h for MH, 12 and 24 h for MT3H in MH). This increase is attributed to the formation of a porous layer of corrosion products on the surface, which results in a rise in the local dielectric constant. Therefore, the aqueous extract of MT3H in MH can penetrate the pores of the copper metal, damaging its surface. We suggest that copper is more active in the products generated in the MT3H in MH mixture, thereby revealing the impact of the C=C bond on copper degradation. This finding is consistent with our previous results.

## 4. Conclusions

A study on the corrosion of copper immersed in gas-phase atmospheres of Methyl Hexanoate (MH) and 25% Methyl Trans-3-Hexenoate in Methyl Hexanoate (MT3H in MH) was conducted. Our ^1^H NMR and GC-MS results suggest that in the gas-phase atmosphere of MT3H in MH, in addition to the biodiesel surrogates, 2-Pentenoic acid, 4-Methyl, Methyl ester, and Butanoic acid, 3-Methyl-2-Methylene, were present during the assay, in contrast to the MH atmosphere, where only the saturated surrogate had contact with copper. We estimated a corrosion rate of copper exposed to HM vapors of 3.81 ± 1.27 μm/year and 5.08 ± 1.27 μm/year for the MT3H in MH mixture. The effect of the C=C double bond on copper was also observable using SEM, where MT3H induced a more visible deterioration on the metallic surface. The result of the electrochemical tests revealed that the corrosion process is similar for both gas-phase atmospheres of biodiesel surrogates, and remains unchanged with increasing exposure time. In both aqueous extracts, the Cdl values decrease over 24 h because of a decrease in the local dielectric constant and an increase in the thickness of the electrical double layer. Nevertheless, the formation of a porous layer of corrosion products on the metal surfaces occurred as a result of the more constant increase in the Cdl value at some points during the experiment with MT3H in MH. This result suggests that copper is more active in the products generated with the MT3H in MH extract, suggesting the impact of the C=C bond on copper deterioration.

## Figures and Tables

**Table 1:** Main products detected via GC-MS analysis of the condensed corrosive media, Methyl Hexanoate (MH) and Methyl Trans-3-Hexenoate in Methyl Hexanoate (MT3H in HM), degraded at the corresponding degradation temperatures during 1000 h.

MH Degradation Temperature = 145 °C		
No.	Product	Structure
1	Propanedioic acid, butyl-dimethyl ester (Dimethyl butylmalonate)	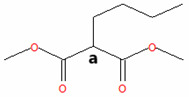
MT3H in MH Degradation Temperature = 158 °C		
No.	Product	Structure
2	2-Pentenoic acid, 4-Methyl, Methyl ester (Methyl 4-methyl-2-pentenoate)	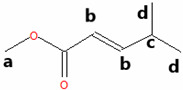
3	Butanoic acid, 3-Methyl-2-Methylene, Methyl ester (methyl 3-methyl-2-methylenebutanoate)	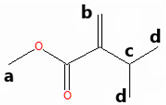
4	4-Pentenoic acid, 2-acetyl-4-Methyl, Methyl ester (methyl 2-acetyl-4-methylpent-4-enoate)	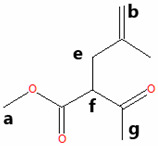

**Table 2:** Electrochemical parameters obtained from the modeling of Figure 5, Figure 6 and Figure 7, considering the equivalent circuit shown in Figure 8.

Time (h)	Rs (Ω·cm^2^)	Rf × 10^4^ (Ω·cm^2^)	CPE_f_		Rct × 10^4^ (Ω)	CPEct		Cdl × 10^−4^ (F·cm^2^)
			*Yf* × 10^−6^ (Ω^−1^·s^n^)	*n*		*Yct* × 10^−5^ (Ω^−1^·s^n^)	*n*	
Distilled water								
0	1545.60	2.45	9.83	0.63	63.50	2.64	0.56	2.37
12	954.81	8.72	981.00	0.70	4.10	30.00	1.00	3.08
18	832.10	16.40	9.20	0.70	98.70	1.46	0.49	2.42
24	953.37	7.57	9.00	0.72	2.01	40.50	1.00	4.05
Methyl Hexanoate								
0	601.62	1.62	16.89	0.54	51.50	7.00	0.72	2.90
12	383.90	1.27	20.52	0.56	70.50	5.63	0.79	1.48
18	377.57	2.34	17.82	0.58	61.80	7.01	0.81	1.75
24	409.83	3.42	16.53	0.59	67.80	8.05	0.85	1.67
25% Methyl Trans-3-Hexenoate in Methyl Hexanoate								
0	435.81	2.44	7.11	0.71	27.20	5.63	0.79	1.18
12	355.35	1.54	21.72	0.62	35.60	6.05	0.80	1.31
18	375.31	3.59	24.43	0.59	40.80	6.01	0.86	1.01
24	341.97	3.47	24.38	0.60	57.50	5.55	0.83	1.14

## Data Availability

The original contributions presented in this study are included in the article. Further inquiries can be directed to the corresponding author.
